# Reliability of digital reactor protection system based on extenics

**DOI:** 10.1186/s40064-016-3618-y

**Published:** 2016-11-10

**Authors:** Jing Zhao, Ya-Nan He, Peng-Fei Gu, Wei-Hua Chen, Feng Gao

**Affiliations:** 1 Shenzhen Institute of Information Technology, Shenzhen, Guangdong China; 2 State Key Laboratory of Nuclear Power Safety Monitoring Technology and Equipment, China Nuclear Power Design CO., LTD, Shenzhen, Guangdong China; 3 Institute of Nuclear and New Energy Technology, Collaborative Innovation Center of Advance-d Nuclear Energy Technology, Tsinghua University, Beijing, China

**Keywords:** RPS, Response time, Extenics, Reliability

## Abstract

After the Fukushima nuclear accident, safety of nuclear power plants (NPPs) is widespread concerned. The reliability of reactor protection system (RPS) is directly related to the safety of NPPs, however, it is difficult to accurately evaluate the reliability of digital RPS. The method is based on estimating probability has some uncertainties, which can not reflect the reliability status of RPS dynamically and support the maintenance and troubleshooting. In this paper, the reliability quantitative analysis method based on extenics is proposed for the digital RPS (safety–critical), by which the relationship between the reliability and response time of RPS is constructed. The reliability of the RPS for CPR1000 NPP is modeled and analyzed by the proposed method as an example. The results show that the proposed method is capable to estimate the RPS reliability effectively and provide support to maintenance and troubleshooting of digital RPS system.

## Bacground

Nuclear safety has been widespread concerned. China has the largest number of NPPs under constructing currently. Along with the implementation of China’s “going out” strategy of nuclear power, the importance of nuclear safety to nuclear power development is self-evident. RPS is directly related to the reliability and safety of NPPs, which has been an important issue to evaluate the safety of NPPs.

RPS is consisted of hardware devices and software components. The interaction of software and hardware determines the reliability of RPS. Normally, the method to analyze the reliability of RPS mainly considers from hardware and software, but it does not take the interaction between hardware and software into account.

Probabilistic safety assessment (PSA) is the main method used to analyze the reliability of RPS’s hardware devices (Ma 2010). PSA is a new accident evaluation method for NPPs developed recently. PSA uses system reliability evaluation techniques (fault tree and event tree analysis) and probabilistic risk assessment techniques to predict the occurrence and progress of various possible accidents in complex systems. PSA mainly focuses on the failure of hardware devices, which does not take the hardware problems caused by software failure into account.

For the reliability analysis of RPS software, the failure mode effect analysis (FMEA) method is putted forward at present (Liu et al. 2015). Software FMEA mainly through identifying the failure mode of software, analyzing the reasons and consequences of failure modes, and taking appropriate measures to eliminate and reduce the harmful consequences, thereby enhancing the reliability of the software. For the software of RPS, there are problems such as failure modes are difficult to be clearly defined, failure probabilities and data are hard to be obtained and need to be isolated from the hardware, when FMEA is used for reliability analysis (He and Shi 2006). Meanwhile FMEA only focuses on the impact of the software itself on the function, which regardless the impact of hardware to achieve the system function.

It is a contradiction that both PSA and FMEA can not solve the problem of software and hardware interaction when computing the reliability of RPS. Extenics is a science to solve the contradiction problem through transformation and expansion. In order to calculate the reliability of RPS, the reliability of RPS and the interaction of hardware and software are needed to be converted. As we all know, the response time of RPS is the result of software and hardware interaction. The software is responsible for the generation of control logic, and the hardware is responsible for controlling the transmission and actions of the instructions. The response time is characterized by the interaction between software and hardware. On the other hand, the reliability of RPS is also characterized. The response time can be regarded as the bridge between software and hardware interaction and RPS reliability.

The paper is organized by five parts as follows. The overall of RPS and its control network model are introduced in the first part. The method to calculate the correlation degree data for each element of the control network model according to extenics correlation function is introduced in the second part. How to establish the reliability model between each element and deduce the calculation method in proposed in the third part. The calculation of the reliability of RPS according to the reliability model established is presented in the fourth part, and the conclusions is given in the last part.

## Overview of reactor protection system

Digital RPS is mainly used to protect the safety of the nuclear reactor, which can ensure reactor trip system to generate reliable and timely protection action in an accident situation, and bring the NPP into a controlled state (Yu et al. 2003).

Generating a complete reactor protection action is a closed-loop control process, which contains four processes, such as generating excitation signals, feeding back of device status, issuing control commands, releasing control signals (Xiao et al. 2013). In some ways, the response time of reactor trip and engineered safety feature (ESF) is related to the reliability of the reactor, meanwhile the response time of each process is directly impacting the safety of reactor.

**Fig. 1:**
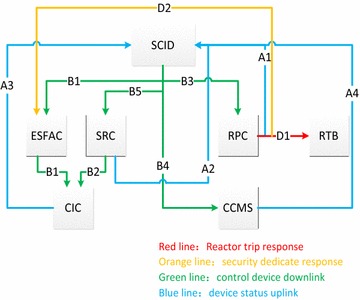
Control network model

In order to establish a reliability relation model of the four steps of the control process, safety bus connections as well as hardwires between cabinets have been simplified to some extent. Cabinets such as core cooling monitor cabinet (CCMS), reactor protect cabinet (RPC), safety related cabinet (SRC) and so on give signals to safety control display cabinet (SCID) about the device status, and encourage it generate and release control commands. The control commands are transmitted to the corresponding cabinet, and the actuators respond to the control actions, then complete a control cycle. Reactor trip instructions and ESF instructions are generated by the output signal of RPC cabinet, which directly acting on reactor trip breaker (RTB) and engineered safety feature action cabinet (ESFAC) carrying out reactor trip and ESF actions. Simplified control network model is shown in Fig. 1.

In the control network model, the blue lines *A*1, *A*2, *A*3 and *A*4 represent uplink paths of the feedback device status. The green lines *B*1, *B*2, *B*3, *B*4 and *B*5 represent the device control command downlink paths. The red line *D*1 represents the reactor trip response route, and the orange line *D*2 represents of ESF response route. It is noted that *B*5 represents device control command downlink path *B*5, meanwhile the downlink path formed by *B*2 and *B*5 represents device control command downlink path *B*2.

It is necessary to be noted that this paper is based on the RPS part of DCS of Yangjiang 5&6 units, but the analysis of the principles and methods can be shared in other types of safety DCS, such as siemens’s TXS and Mitsubishi Electric Corporation’s MELTAC. The structure for DCS of different reactor type will be different in signal transmission path and function distribution. The method proposed in this paper mainly suitable for CPR1000. Since the ACPR1000 is an advanced reactor type which increased some improvements based on CPR1000 after the Fukushima accident, this method is equally applicable. For other reactor types, it is necessary to adjust some technical parameters and model frameworks when using this method.

## Establish reliability model

From the perspective of the response time to analyze the reliability of nuclear reactor, RPS mainly takes reactor trip response time, ESF response time, device control signal downlink time and device status feedback signal uplink time into consideration (Zhou et al. 2013). We know that response time is not the sooner the better normally, and sometimes an abnormal response time indicates there may be a fault or functional failure in somewhere.

In this paper, in order to define the reliability degree of safety–critical system data, we note the measurements of system response test results of safety DCS as *C*, the best theoretical value as *M*, and the worst theoretical value as *N*. *M* is defined as the center point of the interval *C* = [2 *M*–*N*, *N*], with reference to the definition of extenics correlation function (Yang and Cai 2000):

1 $$ K\left( x \right) = \left\{ {\begin{array}{*{20}l} {\frac{2(x - a)}{b - a},} \hfill &\quad {x \le \frac{a + b}{2}} \hfill \\ {\frac{2(b - x)}{b - a},} \hfill &\quad {x \ge \frac{a + b}{2}} \hfill \\ \end{array} } \right. $$

As we know, it means *C* is a bad value and does not reliable, when *C* is less than *M*. Therefore we define the reliability correlation function as below:

2 $$ K\left( C \right) = \left\{ {\begin{array}{*{20}l} {0,} \hfill &\quad {C \le M} \hfill \\ {\frac{N - C}{N - M},} \hfill &\quad {C \ge M} \hfill \\ \end{array} } \right. $$

Then we calculate the correlation degree *K*(*C*), and note it as *K*_*c*_ according to the definition of correlation function. If the measured data is closer to the best value, the correlation degree will be closer to 1, which means the higher reliability degree of the measurement data. On the contrary, if the measured data closer to interval endpoints, the correlation degree will be closer to 0, which means the lower reliability degree of the measurement data.

In order to improve the calculation accuracy of the reliability in RPS, the determined values of M and N are very important, and two methods can be used to determine the specific values of M and N. The first method obtains the values from multiple test data of multiple identical power plants with the same reactor type. This method is obtained in actual operation of power plant, and the data provided from which are more reliable. The other gets the optimal value and the worst value of the whole by theoretical calculating, which compute the optimal value and the worst value of each link. The data obtained by this method may be different from the data obtained in the actual power plant operation. The data of M and N in Tables 1, 2, 3 and 4 of this paper are obtained by the first method, which is analyzing the data of CPR1000 power plant, and lead to a result closer to the real situation of the power plant. The C value is a true measurement, reflecting the current state of operation of the equipment, which can be monitored during the operation of the plant and regularly test to obtain.

**Table 1:** Reactor trip response time

No	Condition	*C* (ms)	*M* (ms)	*N* (ms)	*K*_*c*_
1	High nuclear flux-source range and ((not P6) and (not P10))	98.2	90	110	0.59
2	High nuclear flux intermediate range and (not P10)	80	90	110	0
3	High nuclear flux (low set point) Power range and	93.9	90	110	0.81

**Table 2:** ESF response time

No	Condition	*C* (ms)	*M* (ms)	*N* (ms)	*K*_*c*_
1	Low-low pressurizer pressure	140.6	130	150	0.47
2	High differential pressure in steam line	138.3	130	150	0.59
3	High containment pressure (max 2)	142.1	130	150	0.40

**Table 3:** Control device downlink time

No	Downlink path	*C* (ms)	*M* (ms)	*N* (ms)	*K*_*c*_
1	SCID → ESF → CIC-A3	252	200	500	0.83
2	SCID → SRC → CIC-A3	214	200	500	0.95
3	SCID → RPC	198	200	500	0.99
4	SCID → CCMS	260	200	500	0.80
5	SCID → SRC	476	200	500	0.08

**Table 4:** Device status feeding back uplink time

No	Uplink path	*C* (ms)	*M* (ms)	*N* (ms)	*K*_*c*_
1	RPC III → SCID	318	300	500	0.91
2	CIC-A3 → SCID	352	300	500	0.74
3	SRC-A4 → SCID	376	300	500	0.62
4	CCMS → SCID	376	300	500	0.62

### Reactor trip response time matrix

Reactor trip response time refers to the interval between the instant for RPC receiving sensor signal and the instant for PRC outputing reactor trip command, when any of the 21 kinds of conditions that can trigger reactor trip occurs (Zheng et al. 2010). In order to facilitate the calculation, three conditions are selected for research, with the assumptions of 90 ms for the best response time and 110 ms for the worst one. We calculate the degree of association according to correlation function formula (2). The results are shown in Table 1.

Reactor trip response time matrix is established based on the results calculated in Table 1, and note *C*1 = |*c*1, *c*2, *c*3| = |0.59, 0, 0.81|.

### ESF response time matrix

ESF response time refers to the interval between the instant for RPC receiving sensor signal and the instant for PRC outputting of ESF command, when any of the 49 kinds of conditions that can trigger ESF action occurs. In order to facilitate the calculation, we select three conditions for research, with the assumptions of 130 ms for the best response time and 150 ms for the worst-one. We calculate the degree of association according to correlation function formula (2). The results are shown in Table 2.

ESF response time matrix is established based on the results calculated in Table 2, and note *C*2 = |*c*4, *c*5, *c*6| = |0.47, 0.59, 0.40|.

### Device control signal downlink time matrix

Device control downlink time is the time that SCID control instruction is transferred to the related cabinet. In order to facilitate the calculation, the best and worst value is set to 200 and 500 ms respectively. The degree of association is calculated according to correlation function formula (2), and the results are shown in Table 3.

Device control signal downlink time matrix is established based on the results calculated in Table 3, and note *B* = |*B*1, *B*2, *B*3, *B*4, *B*5| = |0.83, 0.95, 0.99, 0.80, 0.08|.

### Device status feedback uplink time matrix

Device status feedback uplink time refers to the transmission time of the cabinet or the field board feedback the device status to the SCID. In order to facilitate the calculation the best and worst value is set to 300 and 500 ms respectively. The degree of association is calculated according to correlation function formula (2), and the results are shown in Table 4.

Device status feedback uplink time matrix is established based on the results calculated in Table 4, and note *A* = |*A*1, *A*2, *A*3, *A*4| = |0.91, 0.74, 0.62, 0.62|.

## Calculation process

### Associated model

In order to calculate the degree of correlation data and derive the reliability of RPS, an association model between each element to characterize the relationship is established. The reliability data is calculated based on the relationship among the elements. In order to facilitate the calculation, this section will establish a simplified model of RPS reliability, and describe the formulas and conversion of data used in the calculation of RPS reliability.

**Fig. 2:**
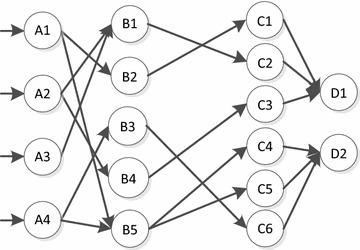
RPS reliability model

The reliability model established shown in Fig. 2, which is used to characterize the relationship of the control process. Matrix *A* = |*A*1, *A*2, *A*3, *A*4| represents reliability of condition signals feedback for field device. Matrix *B* = |*B*1, *B*2, *B*3, *B*4, *B*5| represents reliability of generating control command when received condition signals. Matrix *C* = |*C*1, *C*2| = |*c*1, *c*2, *c*3, *c*4, *c*5, *c*6| represents reliability of control commands issued. Matrix *D* = |*D*1, *D*2| represents reliability of control actions, in which *D*1 represents of reliability of reactor trip action, and *D*2 represents of the reliability of ESF action. The relationship of matrix *A*, *B*, *C*, *D* is shown in Fig. 2.

### Contribution factor

In order to calculate reliability of the entire network, it is necessary to define the contribution degree of each node to the next node, for example the reliability of path that through node *B*5 determined by the reliability of node B5 as well as the reliability of node *A*1 and *A*4 (Hou and Chen 1999). The reliability of node *B*5 is determined by the correlation function. The contribution of *A*1 and *A*4 to *B*5 depends on their importance. If it is assumed that the paths *A*1 and *A*4 are equally important, the contribution factor will be 0.5.

Note the contribution of *Ai* to *Bj* as *Ab*_*ij*_, *Bi* to *Cj* as *Bc*_*ij*_, *Ci* to *Dj* as *Cd*_*ij*_,thus we establish correlation matrix *Ab*, *Bc*, *Cd* of matrix *A*, *B*, *C*, *D*. If the reliability of a node is related to n nodes upstream, the reliability contribution of each node upstream to this node is 1*/n*, thereby the correlation matrix is obtained:

3 $$ Ab = \left| {\begin{array}{*{20}l} { 0,Ab_{12} ,0,0,Ab_{15} } \hfill \\ {0,0,0,Ab_{24} ,0} \hfill \\ {Ab_{31} ,0,0,0,0,} \hfill \\ {0,0,Ab_{43} ,0,Ab_{45} } \hfill \\ \end{array} } \right| = \left| {\begin{array}{*{20}l} { 0,1,0,0,1/2} \hfill \\ {1/2,0,0,1,0} \hfill \\ {1/2,0,0,0,0,} \hfill \\ {0,0,1,0,1/2} \hfill \\ \end{array} } \right| $$

4 $$ Bc = \left| {\begin{array}{*{20}l} {0,Bc_{12} ,0,0,0,0} \hfill \\ {Bc_{21} ,0,0,0,0,0} \hfill \\ {0,0,0,0,0,Bc_{36} } \hfill \\ {0,0,Bc_{43} ,0,0,0} \hfill \\ {0,0,0,Bc_{44} ,Bc_{45} ,0} \hfill \\ \end{array} } \right| = \left| {\begin{array}{*{20}l} {0,1,0,0,0,0} \hfill \\ {1,0,0,0,0,0} \hfill \\ {0,0,0,0,0,1} \hfill \\ {0,0,1,0,0,0} \hfill \\ {0,0,0,1,1,0} \hfill \\ \end{array} } \right| $$

5 $$ Cd = \left| {\begin{array}{*{20}l} {Cd_{11} ,0} \hfill \\ {Cd_{21} ,0} \hfill \\ {Cd_{31} ,0} \hfill \\ {0,Cd_{42} } \hfill \\ {0,Cd_{52} } \hfill \\ {0,Cd_{62} } \hfill \\ \end{array} } \right| = \left| {\begin{array}{*{20}l} {1/3,0} \hfill \\ {1/3,0} \hfill \\ {1/3,0} \hfill \\ {0,1/3} \hfill \\ {0,1/3} \hfill \\ {0,1/3} \hfill \\ \end{array} } \right| $$

It is necessary to be noted that the calculation of contribution factor for a node is mainly concerned with three aspects:

- The importance of the transmission path. The paths transmit the signal for safety equipment is more important than for non-safety equipment.
- The importance of the transmitted signal. The signal used for reactor trip is more important than for ESF.
- The number of transmission signals.

In this paper, the transmission path and signals are assumed to be the same importance, the contribution factor of nodes are measured by the number of transmission signals.

### Numerical relationship

The reliability of the RPS is noted as *K*. Since node *D*1 and *D*2 are output paths to the entire model, the reliability of *D*1 and *D*2 represents the reliability of the entire model. The reliability of *D*1 depends on *D*1, *C*1, *C*2, *C*3 where the reliability of *D*1 is (*C*1*Cd_11_ + *C*2*Cd_21_ + *C*3*Cd_31_)*D1, which can be expressed as (C*Cd).*D by matrix. The node reliability considering the contribution of previous node is noted as $A'$, $B'$, $C'$, $D'$, thus:

6 $$ A' = A $$

7 $$ B' = (A'*Ab).*B $$

8 $$ C' = (B'*Bc).*C $$

9 $$ D' = (C'*Cd).*D $$

10 $$ K = \det (D') $$

Substituting (6), (7), (8), (9) to (10), we get the reliability formula of the entire model:

11 $$ K = det(D') = det((((((A*Ab).*B)*Bc).*C)*Cd).*D) $$

### Model application

According to the control network model (Fig. 1), the signal flow of reactor trip response and ESF response is sorted out, which is shown in Fig. 2. *A*1, *A*2, *A*3 and *A*4 represent the uplink paths which feeding control status back. *B*1, *B*2, *B*3, *B*4 and *B*5 represent the control signal downlink paths. *C*1, *C*2 and *C*3 represent reactor trip response, while *C*4, *C*5 and *C*6 represent ESF response. *D*1 represents the reactor trip action, and *D*2 stands for ESF action (He and Shi 2006).

When the reactor trip condition or ESF condition occurs, device status signal will be feedback via the uplink route *A*1. Then SCID releases control commands through downlink route *B*2, which would result in the reactor trip response and ESF response. It controls the related device to generate reactor trip and ESF action.

According to the results calculated in “Establish reliability model” section, we get matrix *A*, *B* and *C*.

Matrix *D* = |*D*1, *D*2| represents reactor trip action and ESF action, which is the result of control command issued. Matrix D is set to *D* = |*D*1, *D*2| = |1, 1|.

According to the formula (11), we get the reliable calculation formula for reactor protection system:

$$ K = det(D') = det((((((A*Ab).*B)*Bc).*C)*Cd).*D). $$

The reliability of RPS is calculated:

$$ K = det\left( {\left| {0.33,\,0.10} \right|} \right) = 0.215 $$

From the results calculated, we can see that the entire RPS reliability is 0.215. Reactor trip reliability is 0.33, which is higher than the ESF Reliability 0.10. The low reliability of node *B*5 causes low reliability of ESF, which led to a lower reliability of RPS. In engineering practice, if we want to improve the reliability of RPS, increasing the reliability of the node B5 is particularly important. If we improve the reliability of the node *B*5 to 0.90 by means, the ESF calculated reliability will be 0.325, compared with 0.10 before optimization significantly improved. Therefore, this method can not only calculate the reliability of RPS but also apply to work in the engineering aspects for fault diagnosis.

## Conclusions

RPS’s control commands generation, transmission and outputting are the results of the combined effect of software and hardware for the entire system. The proposed method can effectively eliminate the separation of hardware and software from the perspective of response time, and provide a rigorous mathematical derivation process. Analyzing the actual running data of station can effectively identify the reactor protection system reliability shortcomings. At the same time, it also can help to improve system reliability sustained and provide a reference for maintenance as well as fault diagnosis.

Due to length limitations, this article only assumes the contribution factor, while the specific method for determining the contribution factor is ignored. It should be noted that these assumptions do not affect the use of the proposed method. In this paper, the reliability of RPS under several operating conditions is discussed, and more work conditions can be added for research. Meanwhile, the method can also be used for other systems reliability analysis, such as the core cooling monitoring system.
